# Alcohol Consumption in Demographic Subpopulations: An Epidemiologic Overview

**DOI:** 10.35946/arcr.v38.1.02

**Published:** 2016

**Authors:** Erin Delker, Qiana Brown, Deborah S. Hasin

**Affiliations:** Erin Delker, M.P.H., is an assistant research scientist at the New York State Psychiatric Institute, New York, New York. Qiana Brown, Ph.D., M.P.H., M.S.W., is a postdoctoral research fellow in the Department of Epidemiology, Columbia University Mailman School of Public Health, New York, New York. Deborah S. Hasin, Ph.D., is professor of epidemiology at Columbia University Medical Center/New York State Psychiatric Institute, New York, New York

**Keywords:** Alcohol consumption, alcohol abuse and dependence, alcohol use disorder, health consequences, burden of health, injury, demographics, epidemiology, age, race, ethnicity, gender, surveys, National Epidemiologic Survey on Alcohol and Related Conditions, National Survey on Drug Use and Health

## Abstract

Alcohol consumption is common across subpopulations in the United States. However, the health burden associated with alcohol consumption varies across groups, including those defined by demographic characteristics such as age, race/ethnicity, and gender. Large national surveys, such as the National Epidemiologic Survey on Alcohol and Related Conditions and the National Survey on Drug Use and Health, found that young adults ages 18–25 were at particularly high risk of alcohol use disorder and unintentional injury caused by drinking. These surveys furthermore identified significant variability in alcohol consumption and its consequences among racial/ethnic groups. White respondents reported the highest prevalence of current alcohol consumption, whereas alcohol abuse and dependence were most prevalent among Native Americans. Native Americans and Blacks also were most vulnerable to alcohol-related health consequences. Even within ethnic groups, there was variability between and among different subpopulations. With respect to gender, men reported more alcohol consumption and binge drinking than women, especially in older cohorts. Men also were at greater risk of alcohol abuse and dependence, liver cirrhosis, homicide after alcohol consumption, and drinking and driving. Systematic identification and measurement of the variability across demographics will guide prevention and intervention efforts, as well as future research.

Alcohol consumption is common across diverse populations in the United States; however, the level of consumption and its consequences vary considerably across major demographic subgroups. This review presents findings on the distribution and determinants of alcohol use and its aspects (i.e., age of onset, abstention vs. any drinking, binge drinking, and heavy drinking), alcohol abuse and dependence as defined in the *Diagnostic and Statistical Manual of Mental Disorders, Fourth Edition* (DSM–IV) (American Psychiatric Association 1994),^1^ and related health consequences. The health consequences considered include a selection of those often linked to alcohol consumption, such as unintentional and intentional injuries as well as liver disease (World Health Organization 2011). The article aims to summarize recent research and provide a comprehensive depiction of alcohol consumption and alcohol-related group differences across age, race/ethnicity, and gender. The growing emphasis on these group differences in alcohol epidemiologic research can expand our understanding of the etiology of alcohol use disorder (AUD), including the contribution of social contextual risk factors, and the receipt of prevention and treatment services.

The information presented in this article is based primarily on self-reported alcohol use as ascertained in two large surveys of the U.S. general population—the National Epidemiologic Survey on Alcohol and Related Conditions (NESARC) and the National Survey on Drug Use and Health (NSDUH). The NESARC, funded by the National Institute on Alcohol Abuse and Alcoholism, with supplemental funding from the National Institute on Drug Abuse, is a two-wave, longitudinal study of adults ages 18 and older that provides rich information on the epidemiology of alcohol and drug use disorders, psychiatric disorders, other health-related conditions and characteristics, and risk and protective factors (Grant et al. 2004). To ascertain these conditions, the survey used the interviewer-administered Alcohol Use Disorder and Associated Disabilities Interview Schedule—DSM–IV Version (AUDADIS–IV) (Grant 1997). Wave 1 was conducted in 2001–2002 and Wave 2 in 2004–2005. The NSDUH, funded by the Substance Abuse and Mental Health Services Administration (SAMHSA), is a national cross-sectional survey conducted annually of people ages 12 and older that is designed to track trends in substance use and other variables and collects data on substance use through self-administered computerized interviews (SAMHSA 2014).

The estimates presented throughout this article were derived across both waves of the NESARC as well as across several years of the NSDUH. Use of both of these datasets gives readers a comprehensive overview of findings from large-scale U.S. surveys on the epidemiology of alcohol consumption. In addition, the NESARC and NSDUH complement one another in several ways:

- Both surveys include adults age 18 and older. In addition, the NSDUH assesses alcohol and other drug use among adolescents (i.e., ages 12–17). Therefore, incorporating information from both surveys presents a picture of alcohol consumption across the life course.
- Test–retest reliability coefficients for AUDADIS–IV alcohol consumption and AUD diagnoses have been shown to be good to excellent (kappa ≥ 0.60) in a wide range of studies in the United States (Canino et al. 1999; Grant et al. 1995, 2003; Hasin et al. 1997) and elsewhere (Chatterji et al. 1997; Vrasti et al. 1998). AUDADIS–IV alcohol dependence also demonstrated fair to very good concordance with a clinician-administered interview (Cottler et al. 1997) and psychiatrist re-interviews (Canino et al. 1999). The alcohol-dependence factor structure was significantly associated with external criterion variables (Grant et al. 2007), offering further support for the validity of the diagnosis. Less reliability and validity information is available on the NSDUH measure of AUD.
- The NSDUH data have been collected annually on a cross-section of the population, thus supplying a different type of information (i.e., yearly trends) that is not captured in the two waves of the NESARC.
- The two waves of interviews of the NESARC respondents 3 years apart constitute a longitudinal study following a large national cohort of people over time. This allows for causal inference, specifically regarding temporality, as well as for estimates of incidence, persistence, and offset when considering determinates of alcohol use and AUD. In contrast, discerning temporal ordering of variables is more difficult in cross-sectional designs, such as that of the NSDUH.

In addition to the NESARC and NSDUH, this article includes other recently published data from peer-reviewed journals to present the most current information and additional relevant research to supplement findings from these surveys.

## Alcohol-Use Epidemiology

In the NESARC Wave 1 sample, approximately 65 percent of respondents reported any past-year consumption and 51 percent reported consuming at least 12 drinks in the past year (Dawson et al. 2004). Further, 17.8 percent and 4.7 percent, respectively, reported symptoms and criteria indicating a diagnosis of lifetime and past-year alcohol abuse, and 12.5 and 3.8 percent, respectively, reported symptoms and criteria indicating a diagnosis of lifetime and past-year alcohol dependence (Grant et al. 2004; Hasin et al. 2007). Similar results were obtained in secondary analyses with the 2002 NSDUH sample, the survey for which data are available that corresponds most closely to the NESARC Wave 1 sample. In the 2002 NSDUH, approximately 88 percent of respondents reported any alcohol consumption in their lifetime and around 70 percent reported past-year consumption (Grucza et al. 2007). Thus, the differences in estimates are slight.

The two-wave study design of the NESARC enabled researchers to make accurate estimates of the incidence and persistence of alcohol abuse and dependence over a 3-year period. Incident cases are those respondents who developed a disorder for the first time in their lives during the specified period (Grant et al. 2009). In the NESARC, 1-year incidence of alcohol abuse was 1.02 percent and 1-year incidence of alcohol dependence was 1.70 percent (Grant et al. 2009). Persistent cases are respondents who met the criteria for a current disorder at Wave 1 and continued to meet these criteria throughout the 3-year period. An analysis of the persistence of alcohol dependence between Waves 1 and 2 of the NESARC indicated that the disorder persisted in 30.1 percent of respondents with alcohol dependence at baseline (Hasin et al. 2011).

The following sections examine alcohol use and its consequences in specific subgroups of the general U.S. population based on age, race/ethnicity, and gender.

## Alcohol Use and Its Consequences in Different Age Groups

In data analyses by age, the NESARC and NSDUH samples frequently have been collapsed into different age groups. NESARC results commonly are presented in four age groups: 18–29 years, 30–44 years, 45–64 years, and 65 years and older. NSDUH results commonly are divided into five age groups: 12–17 years, 18–25 years, 26–35 years, 36–49 years, and 50 years and older. For clarity, the specific age groups analyzed are clearly identified below when presenting published findings.

More generally, the population can be subdivided into adolescents, young adults, middle-aged adults, and older adults; accurate information on drinking behaviors and related consequences is important for each of these groups. Among adolescents and young adults, alcohol consumption from an early age can have long-term effects on the trajectory of drinking and health consequences across the life course (Patrick et al. 2013); moreover, these two age groups represent the peak age of onset for AUD (Hasin et al. 2007). Middle-aged adults are important to study because many people whose AUD began in young adulthood “mature out” of such a disorder in this age group (Dawson et al. 2005, 2006; Lee et al. 2013; Watson and Sher 1998); further, the mean age of individuals with AUD is 42.2 years (Cohen et al. 2007). Finally, it is essential to examine alcohol use in older adults, because alcohol consumption in this age group can exacerbate many pre-existing vulnerabilities to physical and mental health problems (Sacco et al. 2009).

### Abstention Versus Drinking and Binge Drinking

Despite the fact that alcohol sales to individuals under age 21 are illegal in the United States, many initiate drinking between the ages of 12 and 14, and the prevalence of alcohol use and binge alcohol use increases sharply as adolescents transition into early adulthood (i.e., ages 18–21) (Faden 2006). Consistent with previous studies (Grant 1997; Grant et al. 2001), early drinking initiation in NESARC participants predicted frequency of binge drinking between Waves 1 and 2 (Hingson and Zha 2009). In the NESARC Wave 2 sample, the risk for binge drinking in the 12 months before Wave 2 was approximately twice as high among respondents with drinking onset at age 16 or younger compared with respondents whose drinking began at age 21 or older (Hingson and Zha 2009). In fact, drinking onset across all adolescent age groups (i.e., age 14 or younger, age 15–16, age 17–18, and age 19–20) was associated with significantly higher odds of binge drinking compared with drinking onset at age 21 (i.e., the minimum legal drinking age) (Grant et al. 2001).

The prevalence of any alcohol consumption peaks among young adults. Thus, 73.1 percent of NESARC Wave 1 respondents ages 18–29 reported drinking in the past year. Further, 21.1 percent of young adults reported drinking heavily (5 or more drinks for men or 4 or more drinks for women) more than once a month, and 11 percent reported drinking heavily more than once a week (Dawson et al. 2004). Among young adults, those enrolled in college drink heavily more frequently than their nonstudent counterparts (Dawson et al. 2004).

After age 30, the incidence and prevalence of alcohol consumption generally decreases gradually with age, particularly after age 65 (Chan et al. 2007). In the 2002 NESARC, respondents ages 30–44 had a 25 percent lower prevalence of any past-year drinking compared with respondents ages 18–29. Respondents ages 45–64 and age 65 and older had a 50 percent and 68 percent, respectively, lower prevalence of any past-year drinking compared with the youngest group (Dawson et al. 2004). In the 2002 NSDUH, lifetime and past-year alcohol-use prevalence among adults age 65 and older was 78 percent and 50 percent, respectively (Moore et al. 2009). In the NESARC Wave 1 sample, the odds of past-year alcohol use were particularly low among respondents age 85 or older (odds ratio [OR] = 0.64) and ages 75–84 (OR = 0.64), compared with a reference group of 65- to 74-year-olds (Moore et al. 2009). More recently, in the 2007 NSDUH sample, 43 percent of adults age 65 and older reported past-year alcohol use (Blazer and Wu 2011). The mean number of drinks per drinking occasion also declines with age. Thus, adults ages 18–34 on average consume more than 2 drinks per drinking occasion, adults ages 35–64 between 1 and 2 drinks per occasion, and adults age 65 and older less than 1 drink per occasion (Chan et al. 2007).

### DSM-IV–Defined Alcohol Dependence and Abuse

In the NESARC, prevalence of current and lifetime alcohol abuse and dependence generally decreased with age (Hasin et al. 2007). A similar pattern was evident for incident AUD (Grant et al. 2009). Age of drinking onset also was a predictor of alcohol dependence and abuse in both the NSDUH and NESARC. Among NSDUH respondents age 21 or older at the time of the interview who had started drinking before age 14, about 15 percent reported an AUD after age 21. Among those who had begun to drink at ages 15–17, ages 18–20, or age 21 and older, in contrast, only 9 percent, 5 percent, and 2 percent, respectively, reported an AUD after age 21 (SAMHSA 2014). In the NESARC, respondents with drinking onset before age 16 had approximately twice the odds of developing alcohol dependence/abuse between Waves 1 and 2 compared with respondents whose drinking began at age 21 or later (Hingson and Zha 2009).

In addition, compared with the oldest age group (i.e., age 50 and older), the odds of incident alcohol abuse and dependence after controlling for NESARC Wave 1 demographic and clinical characteristics were significantly higher among people ages 20–29, with ORs of 11.6 for alcohol abuse and 8.7 for alcohol dependence. The risk also was higher among respondents ages 30–54 compared with people age 55 and older (OR = 4.3 for alcohol abuse and OR = 3.5 for alcohol dependence) (Grant et al. 2009). Overall, in the NESARC, 1.2 percent of women and 4.8 percent of men age 50 and older were classified as having either current alcohol dependence or current alcohol abuse (Balsa et al. 2008). Similarly, in the 2005–2007 NSDUH, 1.9 percent and 2.3 percent of adults ages 50–64 endorsed dependence and abuse, respectively, as did 0.6 percent and 0.9 percent, respectively, of adults ages 65 and older (Blazer and Wu 2011).

People in older age groups not only have lower prevalence of alcohol abuse or dependence but also have fewer alcohol-related role-function problems (e.g., problems at work or school). Thus, in the NSDUH, adults ages 26–34 had higher odds of such problems compared with adults ages 65 and older, followed by young adults ages 18–25 and adults ages 35–49, respectively (Alameida et al. 2010).

The finding that younger cohorts were at a higher risk of AUD in both surveys could indicate a true age effect or could be the result of underrepresentation among older cohorts as a result of differential mortality or poor recall of remote events. Birth cohort effects, or historical effects, also may contribute to the observed findings, but prospective population-based investigation is required to adequately address this issue.

### Alcohol-Related Health Consequences

The health burden associated with alcohol use stretches across the lifespan, beginning in utero, with prenatal alcohol exposure resulting in a variety of adverse birth effects, including fetal alcohol syndrome as the most severe consequence (Warren et al. 2011). Over the life course, alcohol use contributes to a variety of health conditions and risk behaviors. Among adolescents, heavy alcohol use is correlated with other risky health behaviors, including tobacco use, violence, suicide, and driving under the influence (Windle 2003). In the NESARC Wave 1 sample, young adults ages 20–29 were most likely to engage in risk behavior after drinking (age 20–24 versus 50 or older, OR = 6.5; age 25–29 versus 50 or older, OR = 4.2) compared with older adults (age 50 or older). The oldest age group (age 50 or older) in the sample was the least likely to drive under the influence of alcohol (Hingson and Zha 2009). Overall, the proportion of alcohol-related deaths was highest among young adults ages 18–24 and decreased with age (Rehm et al. 2014).

## Alcohol Use and Its Consequences in Different Racial/Ethnic Groups

In analyses of NESARC data, alcohol consumption and AUD most commonly have been investigated in five U.S. Census–defined racial/ethnic groups: Whites, Blacks, Native Americans, Asians, and Hispanics. The NSDUH uses the same racial/ethnic categories, with the addition of respondents reporting two or more races, because over time, individuals are increasingly endorsing more than one race, indicating a growing population of people identifying as biracial or multiracial (Hirschman et al. 2000; Jones and Bullock 2012).

### Abstention Versus Drinking and Binge Drinking

In the 2007 NSDUH, current (i.e., past 30 days) alcohol consumption was most prevalent among Whites (59.8 percent) and least prevalent among Asian Americans (38.0 percent). Native Americans/Alaskan Natives (47.8 percent), Hispanics (46.3 percent), and Blacks (43.8 percent) reported similar prevalence of any alcohol consumption (Chartier and Caetano 2010). In the NESARC Wave 1, the prevalence of current alcohol consumption was highest among Whites (63.5 percent), followed by Hispanics (60.3 percent) and Blacks (52.5 percent) (Caetano et al. 2010). However, the prevalence of weekly drinking (i.e., once per week or more) was higher among Hispanics (14.1 percent) than among Whites (13.6 percent) and Blacks (11.4 percent) in the same sample (Caetano et al. 2010).

An analysis of Asian-American adults from the NESARC Wave 2 sample showed that Asians reported the least amount of drinking compared with other groups. However, heterogeneity in alcohol consumption existed within this group, with Korean, Japanese, Taiwanese, and Chinese subpopulations reporting the highest per-capita annual alcohol consumption and Vietnamese, Malaysian, Indian/Afghan/Pakistani, and Indonesian groups reporting the lowest consumption (Cook et al. 2012). The level of acculturation, measured by the use of the subject’s native Asian language, also influenced patterns of alcohol consumption. Among Asian Americans from countries of origin with low per-capita annual alcohol consumption, the probability of being a current drinker was highest among those who reported low use of Asian languages. Among Asian Americans from countries of origin with higher per-capita annual alcohol consumption, the probability of being a current drinker was similar regardless of Asian-language use (Cook et al. 2012).

Hispanic subgroups also display heterogeneity in alcohol consumption. In the 2003–2005 NSDUH, the prevalence of current alcohol use was highest among Cubans, followed by Puerto Ricans, Mexicans, and people of Central/South American descent (Lipsky and Caetano 2009). These patterns differed for binge and heavy drinking, which had the highest prevalence among Puerto Ricans, followed by Mexicans, Cubans, and Central/South Americans. Varying degrees of acculturation may help to explain these subgroup differences among Hispanics; however, the impact of acculturation on drinking also may vary by gender and age (Lipsky and Caetano 2009).

Racial/ethnic differences also exist with respect to binge drinking and heavy drinking during pregnancy. Pregnant White women reported more binge drinking during pregnancy than other racial/ethnic groups (Caetano et al. 2006). However, another study using the Pregnancy Risk Assessment Monitoring System (2001–2005) found that among those who binge drank in the last month, Black, Hispanic, and Asian women were less likely to reduce heavy drinking during pregnancy compared with White women (Tenkku et al. 2009). More research on alcohol consumption patterns among pregnant women by ethnic group is needed to better elucidate racial disparities in the risk for fetal alcohol syndrome (Tenkku et al. 2009).

### DSM-IV–Defined Alcohol Dependence and Abuse

Both alcohol abuse and alcohol dependence are most prevalent among Native Americans and least prevalent among Blacks and Asians. For example, among Native Americans in the NESARC Wave 1 sample, 5.8 percent met criteria for past-year alcohol abuse and 6.4 percent met criteria for past-year alcohol dependence, whereas among Asians, 2.1 percent met criteria for past-year alcohol abuse and 2.4 percent met criteria for past-year alcohol dependence (Hasin et al. 2007). Among Blacks, the prevalence for past-year alcohol abuse and dependence was 3.3 percent and 3.6 percent, respectively, and among Hispanics it was 4.0 percent for both past-year abuse and dependence (Hasin et al. 2007). Among drinkers, Blacks and Hispanics reported more symptoms of past-year alcohol dependence than did Whites (Mulia et al. 2009).

One-year incident rates of alcohol abuse and dependence in the NESARC Wave 2 sample varied little by race (Grant et al. 2009). However, this analysis did not include Native Americans or Asians because of small sample sizes. The only significant difference by race was that Blacks had significantly lower odds than Whites to report incident alcohol abuse (OR = 0.6) at Wave 2 of the NESARC, controlling for Wave 1 demographic characteristics and psychiatric disorders. No significant differences existed between Hispanics and Whites (OR = 0.8) (Grant et al. 2009).

A more recent analysis of Asians within the NESARC Wave 1 sample demonstrated some variations in the lifetime prevalence of AUD among Asian-American ethnic subgroups. For example, 5.4 percent of East Asians (i.e., whose countries of origin were the People’s Republic of China, Japan, Korea, or the Republic of China [Taiwan]), 4.3 percent of Southeast Asians (i.e., whose countries of origin were Indonesia, Malaysia, Vietnam, Thailand, Laos, Cambodia, Myanmar, or a Pacific Island nation), and 3.6 percent of South Asians (i.e., whose countries of origin were India, Afghanistan, Pakistan, or Iran) met criteria for a DSM–IV AUD (Lee et al. 2015).

Among Hispanic subgroups, the prevalence of alcohol abuse and dependence was highest in Mexicans, followed by Puerto Ricans, and was lowest among Cubans (Lipsky and Caetano 2009). Some Hispanic subgroups exhibited a protective effect of foreign-born nativity on risk for alcohol abuse or dependence. For example, in NESARC Wave 1, 4.8 percent of foreign-born Cuban Americans reported a lifetime DSM–IV AUD, compared with 28.1 percent of U.S.-born Cuban Americans. A similar, albeit less extreme, pattern was found among Puerto Ricans, with 14.5 percent of island-born Puerto Ricans but 21.4 percent of U.S.-born Puerto Ricans reporting a lifetime AUD (Alegria et al. 2006).

### Alcohol-Related Health Consequences

The burden of alcohol consumption and AUD on physical health varies by racial/ethnic group. Hispanic White males have higher age-adjusted death rates from liver cirrhosis than non-Hispanic White males, Hispanic Black males, non-Hispanic Black males, and females (i.e., Hispanic White females, non-Hispanic White females, Hispanic Black females, and non-Hispanic Black females) (Yoon and Yi 2012). Within the Hispanic subgroup, Puerto Ricans and Mexicans have the highest mortality rates attributable to liver cirrhosis. Conversely, Asians had the lowest death rates attributable to alcoholic liver disease of all racial/ethnic groups (Hoyert and Xu 2012).

Genetic factors may contribute to racial/ethnic differences in alcohol-related health consequences. For example, in Asian populations, including Asian Americans (Cook et al. 2005; Duranceaux et al. 2008), the prevalence of certain genetic variants encoding the alcohol-metabolizing enzymes alcohol dehydrogenase (ADH) and acetaldehyde dehydrogenase 2 (ALDH2) is higher than in other U.S. racial/ethnic groups. One genetic variant encoding an inactive ALDH2 enzyme that is found primarily in Asian populations is associated with an elevated risk of cancer and digestive disease from alcohol consumption (Oze et al. 2011). This association may apply to Asian Americans as well, a topic warranting further research.

The prevalence of accidents and injuries associated with alcohol consumption, especially with heavy drinking and AUD, also often varies across racial/ethnic groups. For example, the National Violent Death Reporting System provides toxicological information on suicide victims based on coroner/medical examiner reports, death certificates, and toxicological laboratory findings. Analyses of these data have shown that fewer non-Hispanic Blacks (25.6 percent) had positive blood alcohol concentrations at the time of suicide compared with Hispanics (40.3 percent) and non-Hispanic Whites (34.3 percent) (Karch et al. 2006).

Alcohol consumption also is associated with violent crimes. In one study, the offender was under the influence of alcohol in 42 percent of violent crimes studied. However, this percentage differed substantially among racial/ethnic groups and was greatest among Native Americans (62 percent), followed by Whites (43 percent), Blacks (35 percent), and Asians (33 percent) (Chartier et al. 2013). Furthermore, although Blacks in the United States have lower prevalence of alcohol consumption, binge drinking, and AUD compared with non-Hispanic Whites, they still had higher prevalence of alcohol-related homicide (Stahre and Simon 2010). Likewise, Blacks reported drinking during an episode of interpersonal violence more often (i.e., in 41.4 percent of cases) compared with Whites (29.4 percent) and Hispanics (29.1 percent) (Chartier et al. 2013).

Racial/ethnic differences also exist in the prevalence of alcohol use in traffic crashes. According to the National Highway Traffic Safety Administration, the prevalence of intoxication among drivers who are fatally injured in car crashes is highest among Native Americans and Hispanics, followed by Whites, Blacks, and Asians (Chartier et al. 2013). Moreover, Native Americans (4.1 percent) and Whites (3.3 percent) report drinking and driving significantly more often than do Asians (1.4 percent), Hispanics (2.1 percent), and Blacks (1.5 percent) (Chou et al. 2006). However, significant heterogeneity regarding alcohol use and traffic crashes exists within Asians subgroups, with Pacific Islanders and Native Hawaiians reporting prevalence of alcohol-related motor vehicle crashes similar to that of Hispanics (Chartier et al. 2013).

In summary, ethnic minorities make up more than one-fifth of the U.S. population (U.S. Census Bureau 2013). Their risk for drinking, AUD, and other alcohol-related consequences differs markedly. Studies consistently find that Native Americans are at particularly high risk for alcohol-related health consequences. However, despite these negative consequences for Native Americans, their impact on alcohol-related health consequences in the U.S. population overall is less pronounced because Native Americans are a relatively small racial group compared with others. Future research is needed on various ethnic and racial groups to better inform the allocation of prevention and intervention efforts.

## Gender-Differences in Alcohol Use and Its Consequences

### Abstention Versus Drinking and Binge Drinking

Among NESARC Wave I participants, 40 percent of women were abstinent in the past year, compared with 32 percent of men. In addition, men reported more drinks per drinking occasion than women (Chan et al. 2007). Likewise, in the 2011 NSDUH, 57.4 percent of men were past-month drinkers compared with only 46.5 percent of women (Wilsnack et al. 2013). Although epidemiologic findings consistently support that men are at increased risk for alcohol consumption, current drinking, and heavy drinking compared with women, this gap is closing in younger cohorts (Keyes et al. 2008, 2010; SAMHSA 2014). As Western social norms continue to shift away from “traditional” gender roles that see women only as homemakers and mothers, women report greater lifetime largest number of drinks consumed in one sitting and greater frequency of binge drinking than they did in earlier surveys, leading to a closing of the gender gap not only in consumption but also in alcohol-related consequences (Keyes et al. 2008, 2010).

Of particular concern regarding drinking among women is alcohol consumption during pregnancy. Any alcohol drinking during pregnancy can be unsafe (Vall et al. 2015). In particular, binge drinking and heavy drinking during pregnancy are harmful to the fetus and have been related to increased risk for fetal alcohol syndrome (Caetano et al. 2006; Vall et al. 2015). In the NESARC Wave 1 sample, about one-third of pregnant women reported drinking during the last year (Caetano et al. 2006). In the combined NSDUH data from 2012 and 2013, the percentage of pregnant women who reported binge drinking and heavy drinking was 2.3 percent and 0.4 percent, respectively (SAMHSA 2014).

### DSM-IV–Defined Alcohol Dependence and Abuse

In the NESARC Wave 1, the prevalence of current (i.e., in the last 12 months) alcohol abuse and alcohol dependence was 6.9 percent and 5.4 percent, respectively, among men and 2.6 percent and 2.3 percent, respectively, among women (Hasin et al. 2007). Also, between NESARC Wave 1 and Wave 2, men had significantly higher odds than women to develop incidents of alcohol abuse (OR = 2.3) and dependence (OR = 2.4), controlling for Wave 1 demographic characteristics and psychiatric disorders (Grant et al. 2009).

Clinicians often consider AUD among women as “telescoped,” with a later onset of alcohol use but shorter times from initiation to dependence and treatment (Keyes et al. 2008). However, in a recent analysis, Keyes and colleagues (2008) found little evidence for a telescoping effect among women in the general population. Further, sex differences in the prevalence of AUD seem to have decreased over time. As a result, younger women may require more targeted prevention and intervention efforts (Keyes et al. 2008, 2011). Current (Brown et al. 2012) and lifetime (Cavanaugh and Latimer 2010) alcohol abuse or dependence were prevalent among pregnant women (Vesga-Lopez et al. 2008), emphasizing the need for targeted interventions among this population (Mitchell et al. 2008). Women who had been pregnant in the past year also were 1.7 times more likely than non-pregnant women to seek treatment for alcohol abuse or dependence in the previous year (Vesga-Lopez et al. 2008).

### Alcohol-Related Health Consequences

Mortality associated with AUD is higher among men than among women (Rehm et al. 2014). For example, with the exception of Native Americans, mortality rates from alcoholic liver disease were at least twice as high among men compared with women (Hoyert and Xu 2012). Gender differences also existed with respect to alcohol-related morbidity. Thus, although alcohol overall contributed to 32 percent of liver cirrhosis cases, the rates differed significantly between men (39 percent of cases) and women (18 percent of cases) (Room et al. 2005).

With regard to alcohol-related accidents and injuries, males were more likely than females to drive after drinking too much in most age and racial/ethnic groups (Chou et al. 2006). Alcohol also contributed to 7 percent of falls, 10 percent of drowning incidents, and 18 percent of poisonings each year, mostly among men, as well as to a greater proportion of self-inflicted injuries among males (15 percent) than among females (5 percent) (Room et al. 2005). Moreover, male gender was a significant risk factor for alcohol-related suicide in all racial/ethnic groups except Native Americans, where alcohol was involved in similar proportions of male and female suicides (Chartier et al. 2013). Overall, the groups reporting the highest rates of alcohol use among suicide victims were Native Americans ages 30–39, Native Americans and Hispanics ages 20–29, and Asians ages 10–19 (Chartier et al. 2013). Finally, alcohol contributed to 24 percent of homicides, with the proportion of alcohol-related homicides higher among males (26 percent) than among females (16 percent) (Room et al. 2005).

## Methodological Issues

Despite the usefulness of using data from two nationally representative surveys to obtain an accurate picture of alcohol use and its consequences in the U.S. population, methodological differences between the two surveys may have contributed to some differences in population estimates (Grucza et al. 2007). For example, the private, self-administered questions in the NSDUH may have elicited some higher prevalence estimates of use than the face-to-face interviews used in the NESARC. However, the NESARC indicates a higher prevalence of AUD, perhaps resulting from the greater number of items that allowed for more in-depth probing of DSM–IV abuse and dependence criteria. Other factors, including response rates, questionnaire structures, and question text also could contribute to different estimates. Although any of these factors may have contributed to differences between the two surveys (Grucza et al. 2007), the largely common findings across the surveys attest to the robustness of the findings to methodological variation.

## Conclusions

In the United States, AUD accounts for a high and potentially preventable proportion of overall disability and mortality. However, the burden of disease related to alcohol use and its consequences differs significantly between population subgroups. The myriad of genetic, social, and environmental risk factors for AUD and their impact in various subpopulations remain to be elucidated. Future epidemiologic studies will include information necessary to prevent and treat alcohol and drug use disorders by identifying factors that increase the risk of these disorders and their persistence in the general population as well as in specific subgroups.

Population-level surveys, such as the NSDUH and the NESARC, are valuable tools to describe the epidemiology of alcohol consumption and AUD in the United States. Although varying methodology may limit comparability and interpretation of estimates between these epidemiologic studies, both surveys were conducted in nationally representative samples with methodological rigor. Consequently, both surveys present a valid depiction of alcohol consumption and related disorders and can offer important information needed to develop evidence-based measures to prevent the onset of AUD and comorbidity, as well as to identify factors that increase the risk of alcohol problems.

A better understanding of the age, race/ethnicity, and gender-based differences in the various alcohol variables discussed in this review would be gained by considering the social, political, and economic context of alcohol use in various populations. These factors are discussed further in other articles in this issue.

## Footnotes

1 *Alcohol Research: Current Reviews* generally uses the term alcohol use disorder (AUD) to denote the full range of disorders, from abuse to dependence, associated with heavy drinking, as specified in the *Diagnostic and Statistical Manual of Mental Disorders, Fifth Edition* (American Psychiatric Association 2013). Exceptions to this policy may be made when referring to studies using other diagnostic criteria. For more detail on the specific criteria used to diagnose the disorders mentioned in this article, readers should consult the original studies cited in the text.
